# From Cancer Epidemiology to Policy and Practice: the Role of a Comprehensive Cancer Center

**DOI:** 10.1007/s40471-021-00280-7

**Published:** 2022-03-21

**Authors:** Robert A. Hiatt, Amanda Sibley, Brinda Venkatesh, Joyce Cheng, Niharika Dixit, Rena Fox, Pamela Ling, Tung Nguyen, Debora Oh, Nynikka R. Palmer, Rena J. Pasick, Michael B. Potter, Ma Somsouk, Roberto Ariel Vargas, Maya Vijayaraghavan, Alan Ashworth

**Affiliations:** 1 Department of Epidemiology and Biostatistics, UCSF, San Francisco, USA; 2 Helen Diller Family Comprehensive Cancer Center, UCSF, San Francisco, USA; 3 Mission Hall UCSF, 550 16th Street, 2nd Floor, San Francisco, CA 94158 USA; 4 Chinese Community Health Resource Center, San Francisco, USA; 5 Division of Hematology/Oncology, UCSF at Zuckerberg San Francisco General Hospital, San Francisco, USA; 6 Department of Medicine, UCSF, San Francisco, USA; 7 Center for Tobacco Control Research and Education, UCSF, San Francisco, USA; 8 Department of Family and Community Medicine, UCSF, San Francisco, USA; 9 Division of Gastroenterology, UCSF, San Francisco, USA; 10 Center for Community Engagement, UCSF, San Francisco, USA; 11 Clinical and Translational Science Institute, UCSF, San Francisco, USA

**Keywords:** Cancer epidemiology, Community engagement, Cancer disparities, Implementation science, Population health, Collective impact

## Abstract

**Purpose of Review:**

Cancer incidence and mortality are decreasing, but inequities in outcomes persist. This paper describes the San Francisco Cancer Initiative (SF CAN) as a model for the systematic application of epidemiological evidence to reduce the cancer burden and associated inequities.

**Recent Findings:**

SF CAN is a multi-institutional implementation of existing evidence on the prevention and early detection of five common cancers (i.e., breast, prostate, colorectal, liver, and lung/tobacco-related cancers) accounting for 50% of cancer deaths in San Francisco. Five Task Forces follow individual logic models designating inputs, outputs, and outcomes. We describe the progress made and the challenges faced by each Task Force after 5 years of activity.

**Summary:**

SF CAN is a model for how the nation’s Comprehensive Cancer Centers are ideally positioned to leverage cancer epidemiology for evidence-based initiatives that, along with genuine community engagement and multiple stakeholders, can reduce the population burden of cancer.

## Introduction

As a discipline, epidemiology is perhaps unique in applying itself to multiple levels of biologic organization from genetic and molecular epidemiology to social epidemiology and multiple subdisciplines in between [1]. In cancer research, epidemiology is a critical foundational tool applied across the cancer continuum from prevention to survivorship, but also through systematic intervention assessment and implementation [2]. It is an applied discipline and as such is oriented to the integration of knowledge into multiple sectors of society where evidence derived from science can have an impact on population health. The focus of this article is on a unique initiative designed to reduce the cancer burden in a defined metropolitan area using evidence largely derived from epidemiologic studies.

## Description of the San Francisco Cancer Initiative (SF CAN)

The San Francisco Cancer Initiative (SF CAN) was established in 2015 to reduce cancer-related morbidity and mortality in the City and County of San Francisco under the sponsorship of the University of California, San Francisco (UCSF) and its Helen Diller Family Comprehensive Cancer Center (HDFCCC) in partnership with the San Francisco Department of Public Health (SFDPH), other health care systems, community groups, and nonprofit organizations [3]. San Francisco is a municipality characterized both by wealth and innovation and by pockets of persistent poverty. It is a well-defined population of manageable size, serving as a “population laboratory” for implementing an integrated systems approach for cancer prevention. SF CAN focuses on the most common cancers for which evidence-based prevention and/or early detection interventions and policies are available and which account for approximately 50% of cancer deaths in San Francisco [4]: breast, prostate, lung and other tobacco-induced cancers, colorectal, and liver cancer.

The SF CAN perspective is one that recognizes fundamental cause theory and the importance of social determinants such as income inequality, structural racism, lack of power, and social isolation in leading to the inequities observed [5], the “causes of the causes”[6]. What sets SF CAN apart from almost all other cancer epidemiology applications in the USA are these features: (1) a recognition that such a complex mission cannot be achieved by any academic institution alone but that partnerships with numerous health care and community-based entities are required with the adoption of a collective impact model and backbone institution (i.e., in this case the HDFCCC) [7]; (2) strong support of the HDFCCC Director who believed in a cancer center’s role in advancing population health; (3) a commitment to raise unrestricted funds and to maintain ongoing support for the effort; and (4) taking care to establish and maintain equal partnerships where organizations and individual leaders defined their own role and provided meaningful guidance. Five interlinked task forces were formed, each addressing one of the City’s most prevalent cancers and each charting a course of action based on population data and the evidence base for intervention. It has taken a population-based, multi-level, transdisciplinary approach [8] with active engagement of the political leadership and integrated epidemiologic cancer research, prevention activities, improvements in cancer health care, and community participation. This report focuses on how the initiative has shifted, persisted, and what has been accomplished since its inception in 2015 [3], including its forced temporary contraction during the COVID-19 pandemic. It describes SF CAN for an epidemiologic audience and for those in positions to transform data into an actionable catalyst for multi-level change.

## Epidemiology of Cancer in San Francisco

Cancer has surpassed cardiovascular disease as the number one cause of death in San Francisco and accounted for an average of 1376 deaths per year over the most recent 5-year period [9]. For comparison, there have been a total of 547 deaths from COVID-19 as of June 2021 in the City [10]. Based on cancer registry data and epidemiologic analysis, four of the most common cancers (breast, prostate, lung, and colorectal cancers) plus hepatocellular (liver) cancer accounted for almost half (49.0%) of the annual observed mortality in the 2014–2018 period [9]. Evidence and recommendations based on epidemiologic studies inform preventive and/or early detection practices for each of these cancers. Hepatocellular cancer was included with the top four because of its high prevalence in Asian Americans, who constitute about one-third of the population of San Francisco. Importantly the epidemiologic picture for these five cancer sites also revealed inequities across race/ethnic subgroups in the City and provided a focus for interventions aimed at reducing these inequities [9].

Cancer incidence, stage at presentation, survival and mortality data for all genders, major race/ethnic groups, and cancer sites are published regularly by the Greater Bay Area Cancer Registry (GBACR), which is part of the National Cancer Institute (NCI) supported Surveillance, Epidemiology, and End-Results (SEER) program [9]. For the most recent period (2014–2018), cancer incidence and mortality for both men and women in San Francisco are somewhat lower than California and the nation. Incidence and mortality counts and rates (Table 1) reveal that there have been 19,907 new cases and 6882 deaths over the last 5 years for the five SF CAN target cancer sites.

**Table 1:** Incidence of and mortality from five leading causes of cancer per 100,000 residents in San Francisco by sex, 2014–2018

	**Incidence**			**Mortality**		
**Type of cancer**	**Men**	**Women**	**Total**	**Men**	**Women**	**Total**
Breast						
Count	**	3028	3028	**	425	425
Rate	**	122.4	122.4	**	15.3	15.3
Lung						
Count	1219	1026	2245	797	642	1439
Rate	51.4	37.8	43.7	34	22.4	27.5
Prostate						
Count	1978	**	1978	357	**	357
Rate	78.9	**	78.9	15.5	**	15.5
Colorectal						
Count	936	799	1735	340	302	642
Rate	37.9	29.7	33.7	14.1	10.3	12.2
Liver						
Count	607	211	818	352	156	508
Rate	23.8	7.6	15.6	14.1	5.4	9.5
All						
Count	10,195	9712	19,907	3678	3204	6882
Rate	415.3	374.7	389.1	154.9	111.7	130.6

**Fig. 1:**
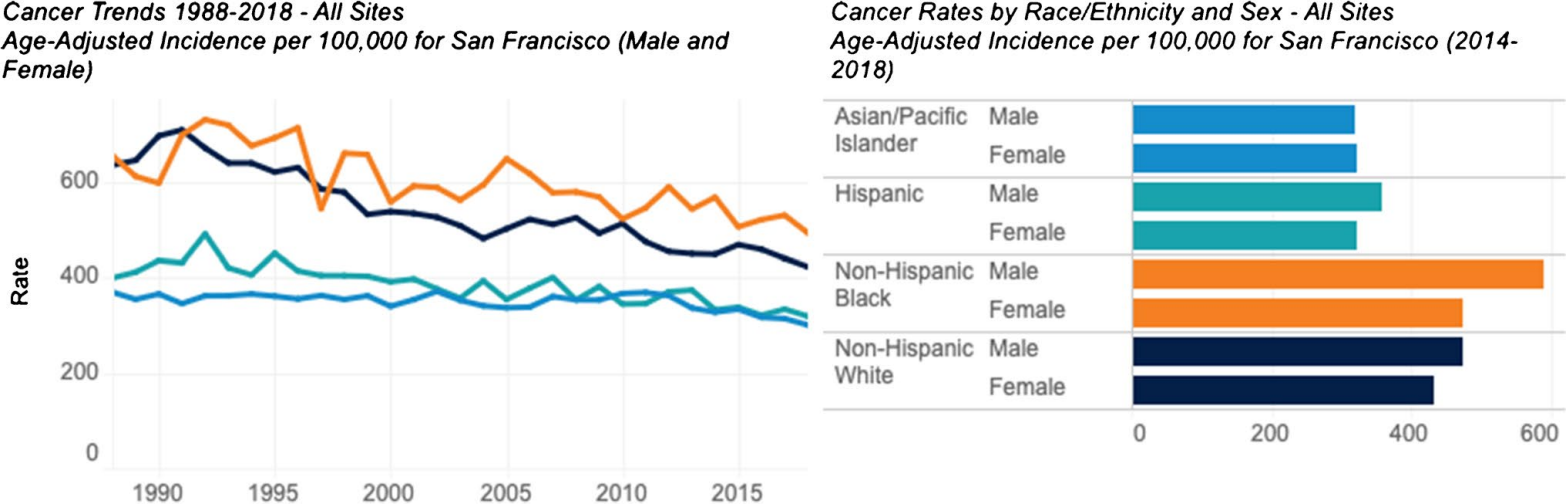
Age-adjusted cancer incidence rates by race/ethnicity and sex in San Francisco

Registry data also reveal large and persistent inequities by race/ethnicity despite an overall decrease in cancer incidence (Fig. 1). In San Francisco, African American residents experience the highest rates of both incidence and mortality for lung, prostate, colorectal, and liver cancer.

**Fig. 2:**
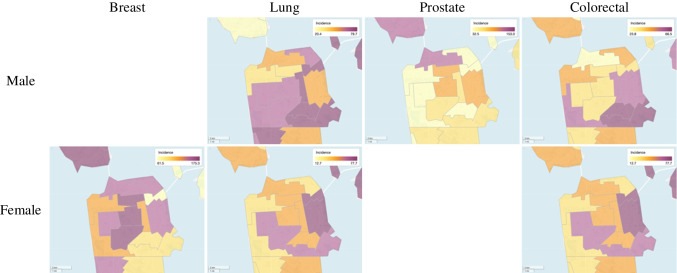
Maps from California Health Maps highlighting highest incidence rates for breast, lung, prostate, and colorectal cancers by neighborhood in San Francisco, 2014–2018

The GBACR developed an interactive mapping tool, California Health Maps [11], using cancer registry data as well as other sources such as the Census and American Community Survey. Using this tool, we can identify zones within San Francisco with the highest incidence rates for our targeted cancers as illustrated for breast, lung, and prostate in Fig. 2. California Health Maps also help characterize sociodemographics in these zones including racial and ethnic composition, socioeconomic status (SES), proportion over age 65 years, and proportion foreign-born. These maps have also highlighted areas with the lowest rates of cancer screening and highest levels of risk factors such as obesity and smoking. These descriptive epidemiologic data have allowed SF CAN to understand the geographic and social context of cancer and to direct its intervention activities.

## Assessing the Needs of the Community

Along with the epidemiologic assessment of the cancer burden in San Francisco, we sought input from our collaborators on the issues and concerns faced by the City’s diverse communities. As previously described [3], we began SF CAN with a series of discussions with representatives of multiple institutions, health systems leaders, community-based organizations, and nonprofit groups to share these descriptive data we had collected in order to elicit interest and involvement as well as to learn about their priorities and how we might benefit from collaborations. Numerous organizations joined SF CAN over the ensuing 5 years as members of the cancer site-specific Task Forces, advisory committees, or project collaborators (see Appendix Table 2). As expected, these coalitions have been dynamic and some original partners have stepped back while new connections formed. Principal among our partners has been the SFDPH, which has the ultimate responsibility for population health in the City, and Kaiser Permanente, a health system that provides cancer care to the largest proportion of San Francisco citizens. Additional information about the needs of the San Francisco community has come from a version of the NCI’s Health Interview National Trends Survey (HINTS) that assessed the level of knowledge and behaviors of a stratified convenience sample of 1,027 (514 preferred English, 256 Spanish, and 257 Chinese [12]) of the most disadvantaged members of the community. This survey sought out individuals who normally do not respond to surveys [13] and 90% of respondents were persons of color who lived in the geographic areas of San Francisco where rates of late-stage cancer incidence and mortality were the highest based on registry data. Analyses of this survey informed us about knowledge and behavioral practices for this population and their preferences for receiving health-related information [12, 14–17].

## Theoretical Framework

SF CAN is theory-driven and began by adopting the PRECEDE-PROCEED model of population behavior change [18] in alignment with existing activities and community goals. This framework generated a process for systematically planning and building the infrastructure for community-based participatory projects, including coalition building and governance. It required the explicit identification of measures of progress as the project proceeded through sequential phases from planning into implementation, and the generation of expected outputs and measurable outcomes. SF CAN is primarily an implementation project focused on the application of what is already known. Each Task Force has developed a logic model to aid in mapping the inputs and expected products and accomplishments on the way to a reduction in the cancer burden and the inequities associated with it. These logic models have been previously published [3].

## Progress to Date

Recognizing that each Task Force’s ultimate impact on decreased inequities in incidence and mortality cannot be measured for several more years, we describe here the successful short- and medium-term progress made and the challenges faced by each Task Force at the 5-year mark.

### Breast Cancer Task Force

The Breast Cancer Task Force aims to increase screening and improve coordination of care after abnormal mammograms for communities and individual women who are in need of support to follow current screening guidelines [19]. In particular, follow-up time after abnormal mammograms is markedly longer for mammography facilities that serve mainly minority and immigrant women when compared to those facilities that serve mainly White women of higher socioeconomic status (SES) [20, 21]. The Task Force has analyzed breast cancer incidence and mortality by neighborhood, race/ethnicity, and SES [22] and used this information to help target particular zones for more intense education and outreach activities. Community outreach is driven by community-based organizations, primary care clinics, and community leaders and supported by the partners in the Task Force. To help facilitate access to existing programs, information on city-wide resources has been collected and published on the SF CAN website in English, Spanish, and Chinese [23]. An important component of this outreach is a mobile mammography unit (MammoVan) that provides convenient and efficient mammography services to women at their own primary care clinics. They also have an ongoing quality improvement (QI) project that leverages QI principles to increase mammography rates and timely follow-up for abnormal mammograms in six primary care clinics that serve low-income residents of San Francisco. In addition, they have trained 29 high school students from targeted communities to disseminate breast cancer and screening information [24].

### Tobacco Task Force

The Tobacco Task Force focuses on primary prevention for lung and other tobacco-related cancers. It aims to reduce smoking among high-risk populations including young adults, people experiencing homelessness, or who live with mental health or substance use disorders. Activities have included efforts to eliminate sales of menthol and flavored tobacco products, which are disproportionately used by youth [25], African Americans [26], and LGBTQ populations [27]. An early success was their provision of research data and advice to the San Francisco Board of Supervisors, which led to the development and unianimous passage of a San Francisco ordinance ending the sale of menthol and flavored tobacco in 2017 [28]. Despite a referendum to repeal the law supported by $12 million from the RJ Reynolds tobacco company, the law was upheld by 68% of voters in 2018 and formally enforced starting in April 2019. Seven SF Bay Area municipalities passed similar policies across the region and the success of these health policy advocacy efforts helped influence the passage of a California state policy to ban flavored tobacco in 2020. The Task Force also focuses on creating smoke-free environments and providing support for smoking cessation for people experiencing homelessness, who smoke at disproportionately high rates [29]. Current efforts are focused on training shelter staff at eight emergency shelters and navigation centers, and pilot testing a medication assistance program in two navigation centers to provide on-site counseling services and access to smoking cessation medications [30].

Finally, young adults have higher tobacco use rates, including menthol cigarette use [31], but few use evidence-based smoking cessation services such as the Quitline [32], which consistently receives only three or fewer calls per month from young adults in San Francisco. The Task Force dramatically increased access for young adults to evidence-based smoking cessation counseling through the launch of 58 smoking cessation groups on social media, enrolling 862 participants over 40 months (average 21.5 per month) with 41% of those completing the program reporting smoking abstinence [29, 30, 33]. In March 2020, the smoking cessation groups were adapted to support adolescents quitting vaping on Instagram in a pilot program that has supported the development of a randomized trial launched in July 2021.

### Prostate Cancer Task Force

The Prostate Cancer Task Force is working to reduce the large inequities in incidence and mortality observed for African American men through targeted early detection and risk-stratified follow-up of aggressive prostate cancer [34]. The Task Force pursues this goal with a multi-level approach to active surveillance and “smarter screening and smarter treatment” (S3T) [35, 36] consistent with current USPSTF recommendations [37, 38]. A Prostate Cancer Action Network (PCAN) has also been established through collaborations with community leaders and primary care and urology leaders from three major health care systems in the City. PCAN annually distributes mini-grants to ten predominantly African American San Francisco churches to foster ongoing participation and education. They also developed a comprehensive community screening protocol that includes navigation for patients with elevated PSAs, educational and digital storytelling videos for health care providers and community members, and launched a bimonthly support group for African American men. Also, a prostate cancer screening and diagnosis algorithm was designed, approved, and implemented in the UCSF Health electronic medical record health maintenance banner and serves as a prototype for other institutions. Ongoing analysis has revealed that these strategies have been associated with a 3-fold increase in the number of African American men receiving a PSA test for the largest of our institutional partners. In addition, the Task Force provided leadership to the California State Cancer Plan 2021–2025 that now encourages the use of risk-stratified screening, diagnosis, and treatment for prostate cancer [39].

### Colorectal Cancer Task Force

The Colorectal Cancer (CRC) Task Force aims to reduce colorectal cancer morbidity and mortality and eliminate inequities through increased screening and detection [38, 40], particularly within uninsured and underinsured populations who receive care in resource-limited clinical settings [41]. They have developed partnerships with the San Francisco Community Clinic Consortium (SFCCC), a group of 12 nonprofit community health centers, and the San Francisco Health Network (SFHN), which operates 12 county-run health clinics. Together these groups provide health care to over 100,000 low-income San Franciscans, as well as many others who live in surrounding counties. With SFCCC, the Task Force has provided resources to support improved reporting of screening rates and led quality improvement initiatives, with educational training, stipends, and technical assistance for individual clinic sites targeting barriers to colorectal screening and diagnostic follow-up that are specific to their patient populations. This has included the creation of new clinic processes and internal policies to support opportunistic screening and novel approaches to navigate homeless patients to colonoscopy when needed [42] and a formal evaluation is underway. With the SFHN, the Task Force has focused on supporting population-based outreach with stool-based screening and navigation to colonoscopy, including pragmatic research on the effectiveness of targeted outreach with text messaging and mail campaigns. Steady improvement in CRC screening rates and follow-up practices at SFCCC and SFHN were observed up until the COVID-19 pandemic began and are now recovering.

The Task Force has developed low literacy educational materials, created patient education videos in multiple languages, and organized meetings to discuss best practices [43, 44]. They also leveraged their local experience to contribute to the California State Cancer Plan and in support of legislative initiatives to address insurance barriers to colorectal cancer screening in California.

### Liver Cancer Task Force

Liver cancer has had among the fastest rising cancer incidence rates and the poorest survival [45]. The Liver Cancer Task Force therefore has a major focus on the prevention, screening, and treatment of Hepatitis C virus (HCV), suppression of Hepatitis B virus (HBV), and improved rates of regular ultrasound exams for persons with chronic hepatitis and cirrhosis. The Task Force provides financial and expert scientific input in a multi-partnership model that includes community organizations, major health care systems, and public health programs. The End Hep C SF coalition [46] is a major partner that provides testing and treatment at public health clinics and trains cured HCV patients as peer navigators to educate untreated HCV patients at community sites such as needle exchange programs [47]. To display the outcomes of this work, End Hep C SF created an online score card displaying results-based accountability indicators such as the numbers of SF HCV–related deaths and hospitalizations; these data show that new HCC diagnoses in SF declined from 86 in 2015 to 69 in 2017. A second partner is the DeLIVER van, a mobile unit that delivers HCV screening and treatment directly to persons who do not have access to traditional medical care, such as those in homeless shelters. Since January 2019, the team has tested hundreds of persons at risk and successfully treated those with confirmed HCV. A third partner is SF Hep B Free, a multi-county campaign to turn the Bay Area into the first HBV-free area in the nation; they provide free and low-cost HBV testing and vaccinations for at risk Asian and Pacific Islander adults.

There are no USPSTF guidelines for liver cancer screening and health systems have either non-existent or poor directives for this neoplasm. In an effort to systematically address this gap, the Task Force targeted UCSF Health and the affiliated Zuckerberg San Francisco General Hospital to investigate metrics of care for HBV and HCV patients. As of 2019, over 35% of 2800 HCV patients had not been treated and of 2500 HBV patients, 45% had never had HBV DNA measured and 38% had never had liver cancer screening. The Task Force is now using the data on these cohorts to help promote the development of sustainable liver disease registries and systematic approaches to treatment in large health systems in San Francisco.

## Discussion

SF CAN is a systematic effort involving a coalition of individuals and institutions from multiple disciplines and sectors faced with a common interest in population health and the reduction of cancer inequities. It has sought to apply sound scientific evidence from epidemiology and other disciplines and to engage committed community organizations, health systems, government, and generous volunteers to make a substantial and sustained impact on the burden cancer represents to San Francisco and its people.

The ultimate goal of reducing cancer mortality will take time; mortality could not be reduced in a few years, even if resources were unlimited. Cancer treatment is improving, but the population health goal must be to make cancer uncommon in the first place through prevention. Overarching the focused work of the Task Forces, SF CAN has sought to address social determinants related to lack of knowledge, social disconnectedness, and access to and quality of care. SF CAN has shown, with the example of the menthol and flavored cigarette ban [28], that it can affect change and policy directed at cancer prevention. Its collaboration with safety net clinics and primary care providers has shown that it can have an impact on policies and procedures that improve the early detection of breast, colorectal, prostate, and liver cancer.

SF CAN is among very few similar efforts underway nationally. We are aware of seven initiatives around the country focused on improving cancer control at the community or population level, three of which have received NCI funding. Most similar to SF CAN is the Be Well Communities program begun by MD Anderson Cancer Center in 2017 to introduce evidence-based prevention interventions in Baytown near Houston starting with children in schools [48]. In Chicago, the Lurie Cancer Center has sponsored the Cancer Health Equity Collaborative since 2015 with the objective of focusing on inequities in prevention and quality care through engaging both community leaders and health care providers [49, 50]. In Boston, the Massachusetts General Hospital and the Kraft Center for Community Health support the Implementation Science Center for Cancer Control Equity, which is focused on community engagement and education, but also on decreasing the financial burden of clinical trial participation [51]. At the Duke Comprehensive Cancer Center in North Carolina, a supplemental grant supports PLACE, a quantitative community health assessment and roadmap for strategic research to reduce cancer disparities and increase and diversify clinical research participation [52]. Other community engagement projects directed at cancer risk factors have relied on philanthropy or public health funding and have not yet been published. These projects include one in New York State, Community Cancer Prevention in Action (CPiA) [53], another the Ohio Colorectal Cancer Prevention Initiative from The Ohio State University Comprehensive Cancer Center [54], and the Latinos United for Cancer Health Advancement (LUCHA) project from the University of California Davis Comprehensive Cancer Center [55]. In contrast to SF CAN, it appears that these interventions focus more on community education and awareness, on a specific subset of the population, or on a specific cancer site rather than on the multi-institutional implementation of evidence-based interventions across multiple major preventable cancers.

Common themes for all these programs are the importance of community engagement and the creation of a “two-way street” leveraging community assets while addressing needs and those of academic, public health, and other institutions and educational efforts to promote awareness of cancer and its prevention, early detection, and treatment. The uniqueness of SF CAN is that it tackles five different common cancer sites in a geographically defined population, uses a theoretical model to advance implementation of proven effective interventions in both prevention and early detection, follows the principles of collective impact, and leverages its efforts by partnerships with ongoing cancer control programs.

A large and complex systems change such as SF CAN also has its challenges. First among these is forming and sustaining a strong coalition and effective governance. We have approached coalition building as described above and have the support of major stakeholders in the city (see Appendix Table 2). However, it is an ongoing process, with new groups continuously introduced into the coalition and others dropping away. We have regular meetings of the Task Forces, the Steering Committee, and the External Advisory Council, and can communicate via the SF CAN website [23], but keeping all stakeholders both informed and involved is a continuing challenge. Integration across Task Force programs is also a challenge since they are targeting different places along the cancer continuum from prevention (tobacco) to early detection (breast, prostate, colorectal) to treatment (liver) and are partnering with different entities including the public health department, safety net clinics, community nonprofits, and advocacy groups. Nevertheless, the common governance and use of logic models keeps the Task Forces grounded in the ultimate goals of SF CAN, while sustaining morale, shared purpose, and financial commitment from the HDFCCC. Resources are and will be challenging to maintain. SF CAN is not a federally funded research project. It is rather an implementation science project for interventions of proven effectiveness [56], using resources from private donations.

Finally, the COVID-19 pandemic has been a major setback to activities within each Task Force either because it has precluded contact with individuals and clinics or because personnel resources have been redirected to fighting the disproportionate burden of disease in the very same population SF CAN is trying to help. Screening rates for mammography, colorectal FIT [57], and PSA testing markedly declined during the pandemic and we expect to see increases in late-stage presentations for breast, colorectal, and prostate cancer as a result. Some of the activities of our staff and investigators have pivoted to providing personal protective equipment, testing, and vaccinations to community members in several settings. In other cases, Task Forces were able to continue activities with special arrangements in place. For example, the Liver Cancer Task Force brought their testing and treatment services to the Shelter in Place hotels and SF Hep B Free created SF Hepatitis B ECHO, a model of centralized specialty care using video conferencing with community clinicians who need help with their HBV patients. Despite these challenges, with sufficient funding SF CAN plans to extend its reach to other preventable cancers, especially those with inequities that disadvantage underserved communities such as human papilloma virus (HPV)–related cancers that can be prevented with the full-scale institution of HPV vaccination.

## Conclusions

SF CAN is an example of how evidence derived largely from epidemiologic studies can be applied to implementation programs and policies to reduce the burden of cancer in a geographically defined population. Few entities are positioned to take the challenge to make real progress in reducing a region’s cancer burden like the nation’s Comprehensive Cancer Centers. The model developed for SF CAN is an engine of innovation for developing creative approaches to community-defined needs for partnership; with appropriate adaption, this model may be implemented in other locales. Challenges persist for sustaining integration and funding for such an endeavor, but it stands as a viable example of how to address the social determinants of cancer and their influences on individual behaviors and group practices.

## Appendix

**Table 2:** List of SF CAN Collaborators (2015-2021)

Organization	Breast	Tobacco	Prostate	Colorectal	Liver	Steering Committee	EAC
Abundant Life Health Ministries			X				
African American Community Health Equity Coalition						X	
African American Tobacco Leadership Council		X	X				
Alameda County Public Health Department			X				
Alto Pharmacy		X					
American Cancer Society	X			X	X		X
Arthur H. Coleman Medical Center			X				
Arthur H. Coleman Community Health Foundation			X				
Asian Pacific Islander Health Parity Coalition						X	
Breast Cancer Connections (Bay Area Cancer Connections)	X						
Breast Cancer Emergency Fund	X						
Breathe CA		X					
California Colorectal Cancer Coalition (C4)				X			
California Department of Public Health			X	X			X
California Dialogue on Cancer (CDOC)				X			
California Smoker’s Helpline		X					
California Urological Association			X				
Cancer Prevention Institute of California	X	X	X	X	X		
Chicano/Latino/Indigena Health Equity Coalition						X	
Chinatown Public Health Center	X						
Chinese Hospital					X		
Circulo de Vida	X						
Colorectal Cancer Coalition (CCA)				X			
Conerstone Missionary Baptist Church			X				
CPMC Sister to Sister Network	X						
CPMC Sutter Health					X		
Dignity Health					X		
End Hep C SF					X		
Fight CRC				X			
Health Right 360 (HR360)				X			
Hope Lab		X					
Jones Memorial United Methodist Church			X				
Kaiser Permanente-San Francisco	X		X	X	X		X
Komen Foundation	X						
Lyon-Martin Health Center				X			
Mission Neighborhood Health Center	X			X			
Missionary Temple Christian Methodist Episcopal Church			X				
National Colorectal Cancer Roundtable (NCCRT)				X			
Neighborhood Baptist Church			X				
Northeast Medical Services				X	X		
One Medical				X			
Operation Access				X			
Pilipino Senior Resource Center	X						
Project Inform					X		
Providence Baptist Church			X				
Public Health Institute, M.E.T.A. Oakland		X					
Rafiki Coalition for Health and Wellness			X			X	
Rescue Agency		X					
Saint Andrew Missionary Baptist Church			X				
Saint Anthony Medical Clinic				X			
Saint James Baptist Church			X				
Saint John Missionary Baptist Church			X				
Saint Paul of the Shipwreck Catholic Church			X				
Saint Phillip Missionary Baptist Church			X				
San Francisco Bay Area Collaborative Research Network				X			
San Francisco Christian Center			X				
San Francisco Community Clinic Consortium	X			X			X
San Francisco Department of Public Health	X	X	X	X	X	X	X
San Francisco Health Improvement Partnership						X	X
San Francisco Health Network	X			X			
San Francisco Health Plan		X		X			
San Francisco Hospital Council							X
San Francisco Tobacco Free Coalition		X					X
San Francisco Veterans Affairs Medical Center					X		
SF Hep B Free					X		
Shanti Margot Murphy Breast Cancer Program	X						
South of Market Health Center				X			
Tabernacle Community Development Corporation			X				
Third Baptist Church			X				
True Hope Church of God in Christ			X				
UCSF	X	X	X	X	X	X	X
UCSF Health			X	X			
Zero Breast Cancer	X						
Zero the End of Prostate Cancer/ZERO360			X				
Zuckerberg San Francisco General Hospital	X	X	X	X	X		
